# iTRAQ-Based Proteomic Analysis of Visual Cycle-Associated Proteins in RPE of *rd12* Mice before and after *RPE65* Gene Delivery

**DOI:** 10.1155/2015/918473

**Published:** 2015-06-01

**Authors:** Qinxiang Zheng, Yueping Ren, Radouil Tzekov, Shanshan Hua, Minghan Li, Jijing Pang, Jia Qu, Wensheng Li

**Affiliations:** ^1^School of Ophthalmology and Optometry, Wenzhou Medical University, Zhejiang 325027, China; ^2^The Roskamp Institute, Sarasota, FL 34243, USA; ^3^Department of Ophthalmology, University of South Florida, Tampa, FL 33612, USA; ^4^Xiamen Eye Center of Xiamen University, Xiamen, Fujian 361005, China

## Abstract

*Purpose.* To investigate the iTRAQ-based proteomic changes of visual cycle-associated proteins in RPE of *rd12* mice before and after *RPE65 *gene delivery. *Mehtods*. The right eyes of *rd12* mice underwent *RPE65* gene delivery by subretinal injection at P14, leaving the left eyes as control. C57BL/6J mice were served as a wide-type control group. ERGs were recorded at P42, and RPE-choroid-sclera complex was collected to evaluate the proteomic changes in visual cycle-associated proteins by iTRAQ-based analysis. Western blot was used to confirm the changes in the differentially expressed proteins of interest. *Results*. ERG parameters improved dramatically at P42 after *RPE65* delivery. The proteomics analysis identified a total 536 proteins with a global false discovery rate of 0.21%, out of which 7 were visual cycle-associated proteins. RALBP-1, RBP-1, and IRBP were reduced in the untreated *rd12* eyes and the former two were improved after gene therapy, confirmed by Western blot analysis. *Conclusions*. *RPE65* gene delivery restored retinal function at P42 and modified the expression of other functional proteins implicated in the visual cycle. The level of RALBP-1 was still below the normal level after gene therapy in *rd12* mice, which may explain the delayed dark adaption in LCA patients undergoing similar therapy.

## 1. Introduction

Vertebrate vision is initiated by the activation of the phototransduction cascade in rod and cone photoreceptor cells of the retina when photons are absorbed by the ubiquitous chromophore 11-*cis*-retinal and converted to its all*-trans*-isomer [1]. Continued function of photoreceptors requires removal of the all-*trans*-retinal and resupply with chromophore [2]. The classical visual cycle regeneration pathway takes place mostly in the retinal pigment epithelium (RPE) and uses a key enzyme, retinoid isomerase, to supply 11-*cis*-retinal for both rod and cone photoreceptors using all-*trans*-retinoid substrates either recycled from photoreceptors as vision byproducts or originating from the choroidal blood supply [3]. The RPE65 protein is the indispensable retinoid isomerase of the canonical RPE visual cycle and it is highly and preferentially expressed in the RPE cells [4–7]. Mutations in the* RPE65* gene cause Leber's congenital amaurosis (LCA), a hereditary retinal degeneration most often transmitted with an autosomal recessive pattern of inheritance [8, 9]. LCA is a blinding disease with an estimated prevalence of about 1 : 80,000 [10]; mutations in more than a dozen genes can cause LCA and RPE65-LCA is thought to represent about 6% of all LCA cases [11].

Retinal degeneration 12 (*rd12*) mouse model is an LCA animal model with a nonfunctional RPE65 protein because of a mutation in the* RPE65* gene [12, 13]. The absence of a functional RPE65 protein interferes with the visual cycle and leads to substantial reduction in 11-*cis*-retinal levels and accumulation of retinyl esters in RPE, which gradually exerts a toxic effect on the retinal photoreceptors and severely affects the visual function. However, histologically* rd12* mice show predominantly cone degeneration while rods appear to be intact with normal expression of rhodopsin and rod transducin at early ages, indicating that it might be the lack of the chromophore 11-*cis*-retinal that leads to a nonrecordable rod ERG response at the early stages of the disease [14]. RPE65-associated LCA recently gained recognition due to the apparent early success achieved in three clinical trials using gene therapy and recombinant adenoassociated virus (AAV) vectors [15–17]. Nine LCA patients received a subretinal injection with an AAV vector and demonstrated partially restored local visual function, with local visual sensitivity improved by ~50-fold in cones and ~63000-fold in rods [15–17]. However, this reconstituted vision cycle was not completely normal but showed slow rod kinetics, resulting in prolonged course of dark-adaptation and decreased visual ability after photobleaching, indicating that the recycling of the retinal chromophore was still abnormal [17]. Currently, most studies are focused on changes in the RPE65 protein, but that may be insufficient to explain this problem. There are several proteins implicated in visual chromophore recycling, but currently there are no reports of changes in these visual cycle-associated proteins as a result of gene therapy.

The present study aims to explore the changes in these proteins after* RPE65* gene delivery, using* rd12* mice, an LCA model caused by a mutation in the* RPE65* gene. In our previous work, we used the same model to explore the proteomic differences occurring in the retina after gene therapy using two-dimensional electrophoresis (2-DE) and mass spectrometry [18]. In this study, we collected tissue samples containing RPE (retina-pigment epithelium-choroid-sclera complex, RPE/Ch/Sc) in* rd12* mice at P42, 4 weeks after AAV subretinal injection. This enabled us to do quantified proteomic study of visual cycle-associated proteins in the RPE, using a more accurate and sensitive technique in protein quantification, the isobaric tagging for relative and absolute protein quantification (iTRAQ) [19, 20]. Seven visual cycle-associated proteins were identified in* rd12* mice. Three of them, RALBP-1, RBP-1, and IRBP, were differentially expressed before and after gene therapy.

## 2. Materials and Methods

### 2.1. Animals

Twelve* rd12* mice (*Rpe65 rd12* or B6(A)-*Rpe65 rd12/J*) were purchased from the Jackson Laboratory (Bar Harbor, ME), and 6 age-matched C57BL/6J mice were obtained from Animal Center of Wenzhou Medical University. All mice were bred and maintained in the Animal Facilities of Wenzhou Medical University. They were kept in a 12-hour light-12-hour dark cycle with an ambient light intensity of 18 lux. All experiments were approved by the Wenzhou Medical University's Institutional Review Board and were conducted in accordance with the ARVO Statement for the Use of Animals in Ophthalmic and Vision Research. Three groups were assigned in the experiment: the treated right eyes of* rd12* mice were set as the treated* rd12* group, the contralateral untreated left eyes were untreated* rd12* group, and both eyes of age-matched wide-type C57BL/6J mice were the normal control group.

### 2.2. Gene Therapy

The scAAV5-smCBA-hRPE65 vector as used in previous studies was used to deliver* RPE65* gene in* rd12* mice, with the same method of subretinal injections at age P14 [18, 21, 22]. Animals were prepared with pupil dilation and general anesthesia. A 30.5-gauge disposable needle was used to make a small incision in the cornea within the pupil area. Then a 33-gauge, unbeveled, blunt needle mounted on a 5 *μ*L syringe (Hamilton Co., Reno, NV) was introduced through the corneal incision to reach the subretinal space in the inferior central region, avoiding touching the lens and penetrating the neuroretina. One microliter of vector suspension (1 × 10^13^ genome containing particles/mL) containing 1% fluorescein was injected slowly in the subretinal space in the right eye of* rd12* mice. The injected retinal area was visualized by fluorescein positive subretinal blebs demarking the retinal detachment and more than 95% retinal detachment indicates successful injection. After injection, 1% atropine eye drops and 0.3% tobramycin-dexamethasone eye ointment (Alcon Laboratories Inc., Fort Worth, TX) were given 3 times a day for 3 days. Animals with any complications, including iris-cornea adhesion, iris or retinal hemorrhage, and lens injury, were excluded from the experiment.

### 2.3. Electroretinograms

Scotopic and photopic ERGs at ages P14 and P42 were recorded. Full-field ERGs were recorded with a custom-built Ganzfeld dome connecting to a computer based system (Q450SC UV; Roland Consult, Wiesbaden, Germany). Six LED stimuli intensities of −35, −25, −15, −5, 5, and 15 cd·s/m^2^ were applied under scotopic conditions, and 2 white LED stimuli intensities (1 cd·s/m^2^, 1.96 cd·s/m^2^) with a background of 30 cd/m^2^ were used under photopic conditions. After dark adaption overnight, scotopic ERG was always recorded between 8 AM and 11 AM, followed by photopic ERG. All testing was performed in a climate-controlled, electrically isolated dark room under dim red light illumination. Systemic anesthesia was achieved by the intraperitoneal administration of a mixture of ketamine (72 mg/kg) and xylazine (4 mg/kg) and 0.5% proparacaine hydrochloride used to ensure full corneal anesthesia. A small amount of 2.5% methylcellulose gel was applied to the eye, and a special Ag/AgCl wire loop electrode was placed over the cornea as an active electrode. Needle reference and ground electrodes were inserted into the cheek and tail, respectively. Recordings were started from the dimmest light intensity to the brightest. Body temperature was maintained by placing the animals on a 37°C warming pad during the experiment.

### 2.4. Protein Sample Preparation

After retinas were dissected from enucleated eyes, the RPE/Ch/Sc complex was extracted, homogenated, and then mixed with 100 *μ*L ice-cold lysis buffer (7 M urea, 2 M thiourea, 4% CHAPS, 2 M TBP, 20 mM Tris-HCL, 1% IEF buffer, 1 mM PMSF, 100 *μ*g/mL DNase, and 100 *μ*g/mL RNase). The supernatant was obtained after centrifugation at 15,000 rpm for 15 minutes at 4°C. Protein concentrations were determined by BCA assay. Equal protein amount (100 *μ*g) in each group sample was applied to conduct the subsequent iTRAQ.

### 2.5. iTRAQ Labeling and Strong Cationic Exchange (SCX) Fractionation

Following the iTRAQ protocol (Applied Biosystems, Foster City, CA), each 100 *μ*g protein was digested with 0.2 mL of a 50 *μ*g/mL trypsin (Promega, Madison, WI, USA) at 37°C for 16 h. Peptides were labeled with isobaric tags 118 (normal control group), 119 (untreated* rd12* group), and 121 (treated* rd12* group) and incubated at room temperature for 2 h. Then the labeled mixtures were dried by vacuum centrifugation, desalted with Sep-Pak Vac C18 cartridge 1 cm^3^/50 mg (Waters, USA), and fractionated using a ShimazuLC-20AB HPLC Pump system (Shimazu, Japan) connected to a strong cation exchange (SCX) column (polysulfoethyl column, 2.1 mm × 100 mm, 5 *μ*m, 200 Å, The Nest Group, Inc. USA). SCX separation was performed using a linear binary gradient of 0–45% buffer B (350 mM KCl, 10 mM KH2PO4 in 25% ACN, pH 2.6) in buffer A (10 mM KH2PO4 in 25% ACN, pH2.6) at a flow rate of 200 *μ*L/min for 90 min, and 30 fractions were collected every 3 min. Each fraction was dried down and redissolved in buffer C (5% (v/v) acetonitrile and 0.1% formic acid solution), and the fractions with high KCl concentration were desalted with PepClean C-18 spin Column (Pierce, USA).

### 2.6. LC-ESI-MS/MS Analysis

Each fraction was resuspended in buffer A (2% ACN, 0.1% FA) and centrifuged at 20,000 ×g for 10 min. In each fraction, the final concentration of peptides was approximately 0.25 *μ*g/*μ*L. Using an autosampler, 20 *μ*L of supernatant was loaded onto a 2 cm C18 trap column (inner diameter 200 *μ*m) on a Shimadzu LC-20AD nanoHPLC. Peptides were eluted onto a resolving 10 cm analytical C18 column (inner diameter 75 *μ*m) that was assembled in-house. The samples were loaded at 15 *μ*L/min for 4 min and eluted with a 44 min gradient at 400 nL/min from 2 to 35% B (98% ACN, 0.1% FA), followed by a 2 min linear gradient to 80% B, maintenance at 80% B for 4 min, and finally a return to 2% B over 1 min.

The peptides were subjected to nanoelectrospray ionization followed by tandem mass spectrometry (MS/MS) in an LTQ OrbitrapVelos (Thermo) coupled in-line to the HPLC. Intact peptides were detected in the Orbitrap. Peptides were selected for MS/MS using the high-energy collision dissociation (HCD) operating mode with a normalized collision energy setting of 45%. Ion fragments were detected in the LTQ. A data-dependent procedure that alternated between one MS scan followed by eight MS/MS scans was applied for the eight most abundant precursor ions above a threshold ion count of 5,000 in the MS survey scan with the following Dynamic Exclusion settings: repeat counts: 2; repeat duration: 30 s; and exclusion duration: 120 s. The applied electrospray voltage was 1.5 kV. Automatic gain control (AGC) was used to prevent overfilling of the ion trap; 1 × 10^4^ ions were accumulated in the ion trap to generate HCD spectra. For MS scans, the* m/z* scan range was 350 to 2,000 Da.

### 2.7. Database Search and Bioinformatics

The resulting MS/MS spectra were searched against the International Protein Index (IPI) mouse sequence databases (version 3.45) with MASCOT software (Matrix Science, London, UK; version 2.2). Protein identification and quantification for iTRAQ experiments were carried out using the ProteinPilot software v3.0 (Applied Biosystems, USA). The Paragon algorithm in ProteinPilot software was used as the default search program with trypsin as the digestion agent and cysteine modification of methyl methanethiosulfonate. The search also included the possibility of more than 80 biological modifications and amino acid substitutions of up to two substitutions per peptide using the BLOSUM 62 matrix. Only proteins identified with at least 95% confidence, or a ProtScore of 1.3, were reported. A 1.3-fold change was used as the benchmark. All proteins that showed significantly altered expression levels went through Ingenuity Pathway Analysis software (IPA) for pathway and network analysis.

### 2.8. Western Blot Validation

Western blot analyses were performed to validate the differentially expressed proteins. Every two samples in each group were mixed in one Western blot experiment, which was repeated for three times. Protein was extracted with lysis buffer and centrifuged to obtain the supernatant and determine the protein content by BCA assay. Each of the protein samples (30 *μ*g) was subjected to SDS-PAGE and then transferred to polyvinylidene difluoride (PVDF) membranes (Bio-Rad, Hercules, CA). The membranes were blocked for 1 h at room temperature with 5% nonfat dried milk in Tris-buffered saline containing 0.1% Tween 20 (TBST, Applygen Gene Technology Corp). Then they were probed overnight with primary anti-RALBP-1 (ab166655, Abcam, MA, USA, 1 : 1000) and anti-RBP-1 (ab154881, Abcam, MA, USA, 1 : 2000), followed by incubation with horse radish peroxidase (HRP) conjugated goat anti-mouse IgG (Santa Cruz Biotechnology, Santa Cruz, CA, 1 : 2500). Then they were developed with an enhanced chemiluminescence detection kit (Pierce Biotechnology, Inc., Rockford, IL). *β*-actin was used as a loading control.

### 2.9. Statistical Analysis

Results for continuous variables with normal distributions are presented as means ± standard deviations (SD). Nonpaired Student's *t*-test was used to compare means between two groups. Statistical analyses were conducted with SPSS 18.0 (SPSS, Chicago, IL, USA), and a two-tailed *P* < 0.05 was considered significant.

## 3. Results

### 3.1. Electroretinography (ERG) Responses

**Figure 1:**
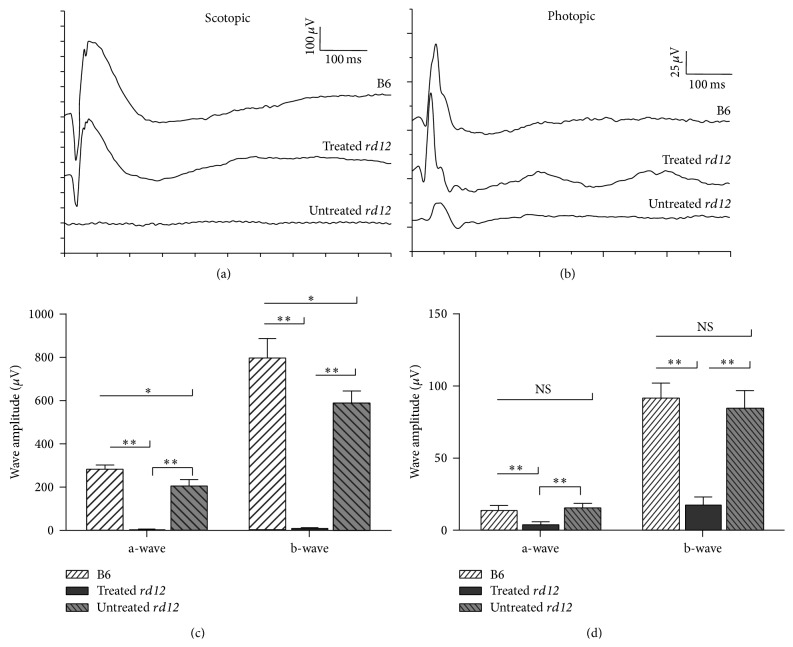
ERG records of untreated* rd12*, treated* rd12*, and normal control C57BL/6J eyes. (a) Scotopic ERG of the 3 groups at P42; (b) photopic ERG at P42. (c) and (d) represent statistical comparison of a-wave and b-wave amplitudes among the different groups at P42 under scotopic and photopic conditions. The untreated* rd12* eyes showed extremely low a-wave and b-wave response in both scotopic and photopic ERGs. The treated* rd12* eyes had great improvement in both a-wave and b-wave amplitudes with normal peak time, close to the wide-type control levels. NS: no significance. ^*^ *P* < 0.05; ^**^ *P* < 0.001.

The untreated* rd12* eyes showed extremely low or even undetectable a-wave and b-wave peaks under scotopic and photopic conditions, indicating severely affected retinal function. Four weeks after AAV injection, a-wave amplitude of the treated* rd12* eyes increased to 210.83 ± 26.70 *μ*V (40-fold of the untreated* rd12* level, *P* < 0.001), close to the 284.50 ± 18.95 *μ*V of the wild-type mice (74.11%, *P* = 0.001) in scotopic ERG with normal peak time; b-wave amplitude increased to 590.00 ± 57.59 *μ*V (48-fold of the untreated* rd12* level, *P* < 0.001), also approaching the 797.67 ± 89.59 *μ*V of the wild-type mice (73.97%, *P* = 0.002) with normal peak time (Figures 1(a) and 1(c)). Photopic ERG signals showed similar trend (Figure 1(b)). Photopic a-wave amplitude of the treated* rd12* eyes improved dramatically to 15.32 ± 2.96 *μ*V (4-fold of the untreated* rd12* level, *P* < 0.001) and was not different from the amplitude recorded from wide-type eyes (13.38 ± 3.43 *μ*V, *P* = 0.363); b-wave amplitude also improved to 84.50 ± 11.99 *μ*V (5-fold of the untreated* rd12* level, *P* < 0.001) and was similar to the wide-type level (91.12 ± 10.62 *μ*V, *P* = 0.377) (Figure 1(d)). This is in accordance with our previous findings [18] and supports the notion that gene therapy could restore retinal function in this animal model.

### 3.2. Identification and Quantitation of Differentially Expressed Proteins after AAV Injection

**Figure 2:**
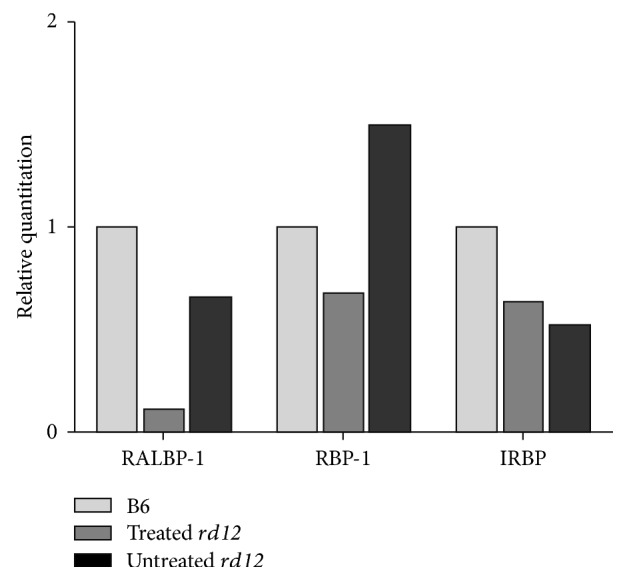
Identification and quantification of differentially expressed proteins by iTRAQ. The RALBP-1, RBP-1, and IRBP were identified as differentially expressed visual cycle-associated proteins among the untreated* rd12*, treated* rd12*, and normal control C57BL/6J eyes. RALBP-1, RBP-1, and IRBP were remarkably reduced in the untreated* rd12* mice (<0.7×) compared to those of C57BL/6J sample. In the treated* rd12* eyes, RALBP-1 was increased to 6-fold of the untreated* rd12* level, although it was still lower than the normal level (0.66-fold); RBP-1 was increased to 2-fold of the untreated* rd12* level and 1.50-fold of the normal level. IRBP level was still lower in the treated* rd12* eyes.

We used iTRAQ proteomics to identify and quantify proteins 4 weeks after* RPE65* gene delivery in* rd12* mice, compared with the untreated* rd12* and normal control C57BL/6J mice. A total of 14432 unique peptides were identified, corresponding to a set of 610 proteins with more than 95% confidence (ProtScore > 1.3, global false discovery rate (FDR) = 0.21%). Of these 610 proteins, 536 were identified with relative quantization, in which 7 were identified as visual cycle-associated proteins including retinaldehyde-binding protein 1 (RALBP-1), retinol-binding protein 1 (RBP-1), interphotoreceptor retinoid-binding protein (IRBP), retinal dehydrogenase 2 (RDH-2), retinal dehydrogenase 5 (RDH-5), lecithin retinol acyltransferase (LRAT), and ezrin-radixin-moesin-binding phosphoprotein 50 (EBP-50) (Table 1). Of these proteins, the expressions of RALBP-1, RBP-1, and IRBP were reduced by at least 0.68-fold in the untreated* rd12* mice relative to the pooled sample of wild-type tissues, while the other four proteins (RDH-2, RDH-5, LRAT, and EBP-50) showed normal levels of expression. In the treated* rd12* eyes, RALBP-1 increased about 6-fold compared to the levels of the untreated* rd12* samples, although it was still lower (66.1%) than the one observed in the samples from wild-type mice. The levels of RBP-1 were also considerably increased in treated* rd12* samples, a 2-fold increase compared to the untreated* rd12* levels and 1.5-fold increase compared to the levels in wild-type mice. In contrast, the expression of IRBP did not increase on P42, and the level in the treated mice was slightly lower (83.2%) compared to the untreated mice (Figure 2). In addition, in the 536 relatively quantified proteins, 91 were downregulated and 71 were upregulated by 1.3-fold in untreated* rd12* eyes compared to the wide-type levels (see Supplementary Tables 1(a) and 1(b) in Supplementary Material available online at http://dx.doi.org/10.1155/2015/918473).

### 3.3. Confirmation of Differentially Expressed Proteins

**Figure 3:**
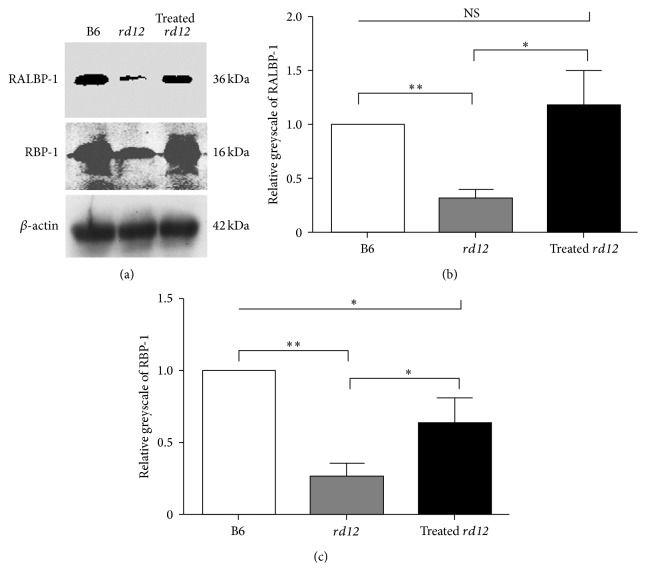
Western blot analysis of differentially expressed proteins. (a) The expression of RALBP-1 and RBP-1 was weak in the untreated* rd12* eyes and appeared to increase significantly following gene therapy to levels similar to those present in normal wild-type control eyes. (b) and (c) show the relative grayscale of RALBP-1 and RBP-1 compared to the wild-type values.

Western blot analysis was performed to validate the changes observed in the differentially expressed visual cycle-related proteins before and after gene therapy. The results confirmed that the expression of RALBP-1 and RBP-1 was much weaker in the untreated* rd12* eyes and that gene therapy increased their level similar to that in the normal C57BL/6J mice (Figure 3).

## 4. Discussion

Gene therapy by subretinal administration of scAAV5-smCBA-hRPE65 vector is a safe and effective treatment to rescue rod and cone photoreceptor function in* rd12* mice, as demonstrated previously [18, 21, 22]. The current investigation focuses on the changes of the visual cycle-associated proteins in* rd12* eyes before and after gene therapy, using the technique of iTRAQ-based 2D LC-MS/MS. Seven visual cycle-associated proteins in RPE layer were identified by this analysis. Three of the seven proteins, RALBP-1, RBP-1, and IRBP, were found to be downregulated in the RPE of* rd12* eyes. Subretinal delivery of* RPE65* by gene therapy demonstrated normalization of the levels of RALBP-1 and RBP-1, while no positive effect was observed on the levels of IRBP.

Visual cycle (or retinoid cycle) is the process by which 11-*cis*-retinal is regenerated from all-*trans*-retinal after photoisomerization, and it mainly takes place in the RPE layer. After conversion from all-*trans*-retinyl ester to 11-*cis*-retinol by isomerase, RALBP-1 acts as an acceptor of 11-*cis*-retinol to produce 11-*cis*-retinal and fulfill the visual cycle [23, 24]. RALBP-1 is another essential protein in the isomerization reaction of the visual cycle, and it plays a critical role to sustain normal retinal function and dark adaptation [25]. Our results demonstrated that the levels of RALBP-1 were dramatically reduced in* rd12* mice, and* RPE65* gene delivery not only regenerated the isomerase RPE65 but also increased the production of RALBP-1, leading to visual cycle restoration and normalization of the visual function. The incomplete recovery of RALBP-1 expression in treated* rd12* eyes may help to explain why the dark adaption was still delayed after similar therapy in LCA patients. Thus, a supplementation of RALBP-1 protein by certain treatment might be a strategy to solve this problem.

IRBP is a large glycoprotein synthesized in the photoreceptors and situated in the interphotoreceptor matrix [26]. It functions as a retinoid-transport vehicle to facilitate the exchange of 11-*cis*-retinal, 11-*cis*-retinol, and all-*trans*-retinol between the RPE, photoreceptors, and Müller cells [27, 28]. Despite some histological and electrophysiological changes because of cytotoxic effects of large amounts of free retinoids, the absence of IRBP in IRBP^−/−^ mice did not cause gross abnormalities in the visual cycle [29]. In this study, the expression of IRBP in* rd12* mice was decreased, which might be attributed to the negatively affected function of the photoreceptors.* RPE65* gene delivery can restore rod and cone function; however, the production of IRBP was not increased and even slightly lower levels were detected compared to pretreatment. One possible explanation for this finding might be that the distribution of IRBP could be influenced by the retinal reattachment after temporary detachment caused by subretinal injection.

RBP-1 is localized in the RPE and serves as a chaperone of all-*trans*-retinol to LRAT in the visual cycle. RBP-1 has been recognized as a pigment epithelium derived factor which supports photoreceptor health and structural integrity [30]. In our results, the production of RBP-1 was reduced in* rd12* mice, suggesting that the RPE function might also be influenced by photoreceptor integrity. Gene therapy could increase the expression of RBP-1 in the RPE substantially, indicating that RBP-1 could resume its function to transport all-*trans*-retinol once the process of retinol recycling is recovered. The other four visual circle-associated proteins including LRAT, RDH-2, RDH-5, and EBP-50 were found to remain at relatively normal expression levels in the RPE of* rd12* mice, demonstrating that not all proteins implicated in vision cycle were affected by the lack of RPE65 function.

The fate of the visual cycle proteins in LCA animal models deserves further investigation since it may reveal unrecognized aspects of the disease process and provide important indications to further improve the visual function in LCA patients after gene therapy. One limitation in this study is that only one time point (P42) after gene therapy was tested. The results could be more informative if earlier (P21) and longer time point (P98) analyses were included. Besides, the dark-adapted ERG seems normal in treated* rd12* eyes, so the ERG after photobleaching could also be tested to illustrate whether the dark adaption course was delayed only after photobleaching, as happened in LCA patients after gene therapy.

In conclusion, our study identified and quantified the RPE levels of visual cycle-associated proteins in* rd12* mice before and after gene therapy.* RPE65* gene delivery restored RPE65 expression and modified the levels of other functional proteins implicated in visual cycle. Gene therapy resulted in incomplete recovery of the levels of RALBP-1 in the RPE of* rd12* mice, indicating that this may also occur in LCA patients undergoing gene therapy and be one of the main causes of observed delayed dark adaption.

## Supplementary Material

Supplementary materials contains:
The supplementary table 1 lists the differentially expressed proteins in untreated rd12 eyes that are up-regulated or down-regulated by 1.3-fold compared to the wide-type levels. Specifically, Table 1A shows 91 down-regulated proteins with an average decrease of 42.7% in the untreated eyes, which was decreased after treatment to 14.0%. Meanwhile, Table 1B presents 71 up-regulated proteins with an average increase of 86.9% in the untreated eyes, which was reduced to 30% after gene therapy with more than 50% improvement.

## Figures and Tables

**Table 1:** The identification and quantification of the visual cycle-associated proteins.

Unused ProtScore	%Cov	Protein name	Peptides (95%)	Untreated *rd12*/B6 (119 : 118)	Treated *rd12*/B6 (121 : 118)
7.74	47	Retinaldehyde-binding protein 1	2	0.1159	0.6607
8.77	68.5	Retinol-binding protein 1	5	0.6792	1.4997
11.16	34.8	Interphotoreceptor retinoid-binding protein	5	0.6368	0.5297
2	22.4	Retinal dehydrogenase 2	1	0.9727	1.1066
41.2	22.4	Retinal dehydrogenase 5	1	1.0864	1.0186
2	63.6	Lecithin retinol acyltransferase	1	0.912	0.9285
8.09	36.9	Ezrin-radixin-moesin-binding phosphoprotein 50	4	0.9638	0.912

## References

[1] Buczyłko J., Saari J. C., Crouch R. K., Palczewski K. (1996). Mechanisms of opsin activation. *The Journal of Biological Chemistry*.

[2] Yang M., Fong H. K. W. (2002). Synthesis of the all-trans-retinal chromophore of retinal G protein-coupled receptor opsin in cultured pigment epithelial cells. *The Journal of Biological Chemistry*.

[3] Bernstein P. S., Law W. C., Rando R. R. (1987). Biochemical characterization of the retinoid isomerase system of the eye. *The Journal of Biological Chemistry*.

[4] Jin M., Li S., Moghrabi W. N., Sun H., Travis G. H. (2005). Rpe65 is the retinoid isomerase in bovine retinal pigment epithelium. *Cell*.

[5] Moiseyev G., Chen Y., Takahashi Y., Wu B. X., Ma J.-X. (2005). RPE65 is the isomerohydrolase in the retinoid visual cycle. *Proceedings of the National Academy of Sciences of the United States of America*.

[6] Redmond T. M., Poliakov E., Yu S., Tsai J.-Y., Lu Z., Gentleman S. (2005). Mutation of key residues of RPE65 abolishes its enzymatic role as isomerohydrolase in the visual cycle. *Proceedings of the National Academy of Sciences of the United States of America*.

[7] Redmond T. M. (2009). Focus on molecules: RPE65, the visual cycle retinol isomerase. *Experimental Eye Research*.

[8] Cideciyan A. V. (2010). Leber congenital amaurosis due to RPE65 mutations and its treatment with gene therapy. *Progress in Retinal and Eye Research*.

[9] Gu S. M., Thompson D. A., Srikumari C. R. (1997). Mutations in RPE65 cause autosomal recessive childhood-onset severe retinal dystrophy. *Nature Genetics*.

[10] Stone E. M. (2007). Leber congenital amaurosis—a model for efficient genetic testing of heterogeneous disorders: LXIV Edward Jackson Memorial Lecture. *American Journal of Ophthalmology*.

[11] den Hollander A. I., Roepman R., Koenekoop R. K., Cremers F. P. M. (2008). Leber congenital amaurosis: genes, proteins and disease mechanisms. *Progress in Retinal and Eye Research*.

[12] Pang J.-J., Chang B., Hawes N. L. (2005). Retinal degeneration 12 (rd12): a new, spontaneously arising mouse model for human Leber congenital amaurosis (LCA). *Molecular Vision*.

[13] Pang J. J., Chang B., Kumar A. (2006). Gene therapy restores vision-dependent behavior as well as retinal structure and function in a mouse model of RPE65 leber congenital amaurosis. *Molecular Therapy*.

[14] Chen Y., Moiseyev G., Takahashi Y., Ma J.-X. (2006). *RPE65* gene delivery restores isomerohydrolase activity and prevents early cone loss in *Rpe65*
^−/−^ mice. *Investigative Ophthalmology & Visual Science*.

[15] Bainbridge J. W. B., Smith A. J., Barker S. S. (2008). Effect of gene therapy on visual function in Leber's congenital amaurosis. *The New England Journal of Medicine*.

[16] Maguire A. M., Simonelli F., Pierce E. A. (2008). Safety and efficacy of gene transfer for Leber's congenital amaurosis. *New England Journal of Medicine*.

[17] Cideciyan A. V., Aleman T. S., Boye S. L. (2008). Human gene therapy for RPE65 isomerase deficiency activates the retinoid cycle of vision but with slow rod kinetics. *Proceedings of the National Academy of Sciences of the United States of America*.

[18] Zheng Q., Ren Y., Tzekov R. (2012). Differential proteomics and functional research following gene therapy in a mouse model of Leber congenital amaurosis. *PLoS ONE*.

[19] Wu W. W., Wang G., Baek S. J., Shen R.-F. (2006). Comparative study of three proteomic quantitative methods, DIGE, cICAT, and iTRAQ, using 2D gel- or LC-MALDI TOF/TOF. *Journal of Proteome Research*.

[20] Wiese S., Reidegeld K. A., Meyer H. E., Warscheid B. (2007). Protein labeling by iTRAQ: a new tool for quantitative mass spectrometry in proteome research. *Proteomics*.

[21] Li X., Li W., Dai X. (2011). Gene therapy rescues cone structure and function in the 3-month-old rd12 mouse: a model for midcourse RPE65 leber congenital amaurosis. *Investigative Ophthalmology & Visual Science*.

[22] Li W., Kong F., Li X. (2009). Gene therapy following subretinal AAV5 vector delivery is not affected by a previous intravitreal AAV5 vector administration in the partner eye. *Molecular Vision*.

[23] Golovleva I., Bhattacharya S., Wu Z. (2003). Disease-causing mutations in the cellular retinaldehyde binding protein tighten and abolish ligand interactions. *The Journal of Biological Chemistry*.

[24] McBee J. K., van Hooser J. P., Jang G. F., Palczewski K. (2001). Isomerization of 11-cis-retinoids to all-trans-retinoids in vitro and in vivo. *The Journal of Biological Chemistry*.

[25] Saari J. C., Nawrot M., Kennedy B. N. (2001). Visual cycle impairment in cellular retinaldehyde binding protein (CRALBP) knockout mice results in delayed dark adaptation. *Neuron*.

[26] Gonzalez-Fernandez F., Kittredge K. L., Rayborn M. E. (1993). Interphotoreceptor Retinoid-Binding Protein (IRBP), a major 124 kDa glycoprotein in the interphotoreceptor matrix of *Xenopus laevis*: characterization, molecular cloning and biosynthesis. *Journal of Cell Science*.

[27] Redmond T. M., Wiggert B., Robey F. A. (1985). Isolation and characterization of monkey interphotoreceptor retinoid-binding protein, a unique extracellular matrix component of the retina. *Biochemistry*.

[28] Betts-Obregon B. S., Gonzalez-Fernandez F., Tsin A. T. (2014). Interphotoreceptor retinoid-binding protein (IRBP) promotes retinol uptake and release by rat Muller cells (rMC-1) in vitro: implications for the cone visual cycle. *Investigative Ophthalmology & Visual Science*.

[29] Ripps H., Peachey N. S., Xu X., Nozell S. E., Smith S. B., Liou G. I. (2000). The rhodopsin cycle is preserved in IRBP 'knockout' mice despite abnormalities in retinal structure and function. *Visual Neuroscience*.

[30] Wang X., Tong Y., Giorgianni F., Beranova-Giorgianni S., Penn J. S., Jablonski M. M. (2010). Cellular retinol binding protein 1 modulates photoreceptor outer segment folding in the isolated eye. *Developmental Neurobiology*.

