# Supplementary material for: iTRAQ-Based Proteomic Analysis of Visual Cycle-Associated Proteins in RPE of rd12 Mice before and after RPE65 Gene Delivery
Source: J Ophthalmol. 2015 Jun 1;2015:918473. doi: 10.1155/2015/918473 (PMC4466473; doi:10.1155/2015/918473)
Supplement: Supplementary file 1 — Supplementary materials contains: The supplementary table 1 lists the differentially expressed proteins in untreated rd12 eyes that are up-regulated or down-regulated by 1.3-fold compared to the wide-type levels. Specifically, Table 1A shows 91 down-regulated proteins with an average decrease of 42.7% in the untreated eyes, which was decreased after treatment to 14.0%. Meanwhile, Table 1B presents 71 up-regulated proteins with an average increase of 86.9% in the untreated eyes, which was reduced to 30% after gene therapy with more than 50% improvement. [file 918473.f1.doc]

Supplementary table 1A. The differentially expressed proteins down-regulated by 1.3-fold identified by proteomic analysis.

| N | Unused Protscore | %Cov | Protein Name | Peptides (95%) | Untreated vs. normal | Treated vs. normal | Effect of treatment |
| --- | --- | --- | --- | --- | --- | --- | --- |
|  |  |  |  |  | (% change) | (% change) | (% change) |
| 1 | 7.74 | 47 | Retinaldehyde-binding protein 1 | 2 | -88.50% | -33.90% | 54.6% |
| 2 | 20.19 | 82.4 | Hba-a2;Hba-a1 hemoglobin alpha, adult chain 2 | 14 | -88.0% | -59.1% | 28.9% |
| 3 | 4 | 53.3 | Sod1 Superoxide dismutase | 2 | -84.7% | -68.1% | 16.6% |
| 4 | 29.52 | 99.3 | Beta-globin | 27 | -81.3% | -84.6% | -3.3% |
| 5 | 8.86 | 33.7 | Lum Lumican precursor | 4 | -81.3% | -73.2% | 8.1% |
| 6 | 5.65 | 38.3 | Apoa1 apolipoprotein A-I | 3 | -79.5% | -73.9% | 5.5% |
| 7 | 4.18 | 46.6 | Gnat1 Guanine nucleotide-binding protein G(t) subunit alpha-1 | 2 | -76.9% | -62.0% | 14.9% |
| 8 | 3.08 | 37.8 | Try4;1810049H19Rik Tesp4 protein | 1 | -74.7% | 219.2% | 293.8% |
| 9 | 3.81 | 81.6 | Dbi Acyl-CoA-binding protein | 1 | -73.5% | -72.0% | 1.5% |
| 10 | 10.13 | 64.2 | Myl6 Isoform Smooth muscle of Myosin light polypeptide 6 | 3 | -73.0% | -81.3% | -8.3% |
| 11 | 4.14 | 38 | Glo1 Lactoylglutathione lyase | 2 | -71.7% | -57.2% | 14.5% |
| 12 | 7.63 | 42.4 | Glul Glutamine synthetase | 3 | -70.9% | 108.9% | 179.8% |
| 13 | 3.86 | 25.9 | Tyrp1 5,6-dihydroxyindole-2-carboxylic acid oxidase precursor | 0 | -66.9% | -22.7% | 44.2% |
| 14 | 12.7 | 51.2 | Parkinson disease (Autosomal recessive, early onset) 7 | 7 | -66.6% | 3.8% | 70.3% |
| 15 | 8.83 | 69.6 | Prdx6 Peroxiredoxin 6 | 4 | -66.3% | -35.1% | 31.1% |
| 16 | 7.83 | 42.9 | Sag S-arrestin | 7 | -64.0% | -65.6% | -1.6% |
| 17 | 36.39 | 83 | Eno1;EG433182;LOC100044223 Alpha-enolase | 22 | -63.4% | -83.1% | -19.7% |
| 18 | 3.29 | 32.9 | Psme1 Proteasome activator complex subunit 1 | 1 | -58.3% | -49.9% | 8.4% |
| 19 | 8.71 | 41.2 | Kera 15 days embryo head cDNA, RIKEN full-length enriched library, clone:D930012M16 product:keratocan, full insert sequence | 5 | -57.9% | -74.9% | -17.0% |
| 20 | 3.69 | 41.7 | 21 kDa protein | 2 | -54.3% | -65.6% | -11.4% |
| 21 | 246.79 | 90.2 | Col1a2 Collagen alpha-2(I) chain precursor | 346 | -53.4% | -1.8% | 51.6% |
| 22 | 195.3 | 69.2 | Col12a1 procollagen, type XII, alpha 1 | 128 | -50.8% | -82.6% | -31.8% |
| 23 | 7.32 | 47.8 | Hnrnpu Osteoclast-like cell cDNA, RIKEN full-length enriched library, clone:I420039N16 product:heterogeneous nuclear ribonucleoprotein U, full insert sequence | 3 | -50.8% | -18.3% | 32.5% |
| 24 | 6.75 | 28.3 | Ctsd B6-derived CD11 +ve dendritic cells cDNA, RIKEN full-length enriched library, clone:F730002E02 product:cathepsin D, full insert sequence | 4 | -49.4% | -84.7% | -35.3% |
| 25 | 1.41 | 22.1 | Nol5a Nucleolar protein 5A | 1 | -49.4% | 28.2% | 77.7% |
| 26 | 4.54 | 23.3 | Hsp90aa1 Heat shock protein HSP 90-alpha | 4 | -48.0% | -48.0% | 0.0% |
| 27 | 63.91 | 60.8 | Ahnak AHNAK nucleoprotein isoform 1 | 32 | -47.5% | -28.2% | 19.3% |
| 28 | 22.7 | 92.3 | Hist1h2bk 14 kDa protein | 14 | -47.5% | 118.8% | 166.3% |
| 29 | 8 | 64.4 | LOC100048062;Rplp2 LOC665931 protein | 6 | -47.5% | -71.2% | -23.6% |
| 30 | 3.3 | 19.3 | Igh-3 Ig gamma-2B chain C region, membrane-bound form | 2 | -47.5% | -31.5% | 16.1% |
| 31 | 1.4 | 16 | Cmas Isoform 1 of N-acylneuraminate cytidylyltransferase | 1 | -47.5% | 13.8% | 61.3% |
| 32 | 35.09 | 64.7 | Atp5b ATP synthase subunit beta, mitochondrial precursor | 25 | -45.1% | -64.7% | -19.6% |
| 33 | 2.4 | 44.1 | Hspe1 10 kDa heat shock protein, mitochondrial | 1 | -45.1% | -44.0% | 1.0% |
| 34 | 2.02 | 17.8 | Atp1b1 Sodium/potassium-transporting ATPase subunit beta-1 | 1 | -44.5% | -80.2% | -35.7% |
| 35 | 40.32 | 63 | Alb Serum albumin precursor | 27 | -43.5% | -82.8% | -39.3% |
| 36 | 8.88 | 85.2 | Mylpf Myosin regulatory light chain 2, skeletal muscle isoform | 6 | -43.0% | -84.2% | -41.2% |
| 37 | 2.82 | 39.8 | Krt5 60 kDa protein | 1 | -41.9% | -32.1% | 9.8% |
| 38 | 2.88 | 29.3 | Pafah1b2 Platelet-activating factor acetylhydrolase IB subunit beta | 1 | -41.4% | -85.6% | -44.2% |
| 39 | 2 | 25.6 | Slc2a1 solute carrier family 2 (facilitated glucose transporter), member 1 | 1 | -41.4% | -84.0% | -42.6% |
| 40 | 1.7 | 13.3 | Lgals1 Galectin-1 | 1 | -40.8% | -55.9% | -15.1% |
| 41 | 1.8 | 11.6 | Ddah1 N(G),N(G)-dimethylarginine dimethylaminohydrolase 1 | 0 | -40.3% | -61.3% | -21.0% |
| 42 | 4.86 | 39.2 | Car2 Carbonic anhydrase 2 | 2 | -39.7% | -52.6% | -12.8% |
| 43 | 2.03 | 64.1 | Hspb1 Isoform A of Heat shock protein beta-1 | 1 | -39.7% | -60.9% | -21.2% |
| 44 | 2 | 52.8 | Hint2 Histidine triad nucleotide-binding protein 2 | 1 | -39.7% | 43.2% | 83.0% |
| 45 | 13.95 | 85.6 | Ppia Peptidyl-prolyl cis-trans isomerase | 6 | -38.1% | 24.7% | 62.8% |
| 46 | 2 | 41.9 | Crocc Isoform 1 of Rootletin | 2 | -38.1% | 17.0% | 55.0% |
| 47 | 9.85 | 44.8 | Got1 glutamate oxaloacetate transaminase 1, soluble | 5 | -36.9% | -40.8% | -3.9% |
| 48 | 4.12 | 41.9 | Cbr1 Carbonyl reductase [NADPH] 1 | 2 | -36.3% | -26.9% | 9.4% |
| 49 | 2.29 | 50 | Ddt D-dopachrome decarboxylase | 1 | -36.3% | -38.6% | -2.3% |
| 50 | 11.16 | 34.8 | Interphotoreceptor retinoid-biding protein | 5 | -36.3% | -47.0% | -10.7% |
| 51 | 1.52 | 41.7 | Hmgn1;LOC100044391 Non-histone chromosomal protein HMG-14 | 1 | -35.7% | 191.1% | 226.8% |
| 52 | 3.59 | 21.2 | Nsf 83 kDa protein | 2 | -35.1% | 7.7% | 42.8% |
| 53 | 12.52 | 41.6 | Trf Serotransferrin precursor | 4 | -34.5% | -53.0% | -18.5% |
| 54 | 4.01 | 17.3 | C3 complement component 3 | 2 | -34.5% | -28.9% | 5.7% |
| 55 | 2.23 | 30.7 | Clec3b C-type lectin domain family 3, member b | 1 | -34.5% | -25.5% | 9.0% |
| 56 | 4.73 | 25.5 | Ganab Isoform 1 of Neutral alpha-glucosidase AB precursor | 2 | -33.9% | 12.7% | 46.7% |
| 57 | 12.44 | 52.8 | Pgk1 Phosphoglycerate kinase 1 | 5 | -33.3% | -66.9% | -33.6% |
| 58 | 1.52 | 13.7 | Dpep1 Dipeptidase 1 precursor | 1 | -33.3% | -31.5% | 1.9% |
| 59 | 8.77 | 68.5 | Retinol-binding protein 1 | 5 | -32.08% | 50.0% | 82.1% |
| 60 | 2 | 12.9 | LOC100048430;Tpt1;LOC100043703 Translationally-controlled tumor protein | 1 | -32.7% | -20.6% | 12.1% |
| 61 | 2 | 32.4 | Acsl6 acyl-CoA synthetase long-chain family member 6 isoform 2 | 1 | -31.5% | -26.2% | 5.2% |
| 62 | 298.76 | 86.7 | Col1a1 Isoform 1 of Collagen alpha-1(I) chain precursor | 447 | -30.8% | -35.1% | -4.3% |
| 63 | 2.92 | 26.1 | 18 kDa protein | 1 | -30.8% | 40.6% | 71.4% |
| 64 | 2 | 33.2 | Rab1 Ras-related protein Rab-1A | 2 | -30.8% | -15.3% | 15.5% |
| 65 | 2.12 | 15.8 | Txndc5 Thioredoxin domain-containing protein 5 precursor | 1 | -30.2% | -61.6% | -31.5% |
| 66 | 2.04 | 72.6 | Rbmx Heterogeneous nuclear ribonucleoprotein G | 1 | -30.2% | 120.8% | 151.0% |
| 67 | 4.55 | 14.5 | Iqgap1 Ras GTPase-activating-like protein IQGAP1 | 2 | -29.5% | -9.6% | 19.9% |
| 68 | 2.09 | 12.3 | Tgfbi Transforming growth factor-beta-induced protein ig-h3 precursor | 1 | -29.5% | -24.1% | 5.4% |
| 69 | 1.84 | 58.9 | Col16a1 Procollagen type XVI alpha 1 | 1 | -29.5% | 18.0% | 47.6% |
| 70 | 1.7 | 30 | Lin7c Lin-7 homolog C | 1 | -29.5% | 369.9% | 399.4% |
| 71 | 1.75 | 70.1 | Hist1h2ba Histone H2B type 1-A | 7 | -28.9% | 324.6% | 353.5% |
| 72 | 4 | 36 | Cyc1 Isoform 1 of Cytochrome c1, heme protein, mitochondrial precursor | 2 | -28.2% | -46.5% | -18.3% |
| 73 | 2.19 | 54.3 | Hnrnpc Isoform 5 of Heterogeneous nuclear ribonucleoproteins C1/C2 | 1 | -28.2% | -6.2% | 22.0% |
| 74 | 5.49 | 49.5 | Prdx2 Peroxiredoxin-2 | 2 | -27.6% | 25.9% | 53.5% |
| 75 | 1.56 | 22.4 | Decr1 2,4-dienoyl-CoA reductase, mitochondrial precursor | 1 | -27.6% | 5.7% | 33.2% |
| 76 | 4.01 | 60.3 | Vamp2 Vesicle-associated membrane protein 2 | 2 | -26.9% | -3.6% | 23.3% |
| 77 | 2 | 28.6 | Dctn1 Isoform 1 of Dynactin subunit 1 | 1 | -26.9% | 64.4% | 91.3% |
| 78 | 9 | 33 | Cilp2 cartilage intermediate layer protein 2 | 4 | -26.2% | 47.2% | 73.4% |
| 79 | 3.52 | 34.3 | EG432834 hypothetical protein | 2 | -26.2% | -24.1% | 2.1% |
| 80 | 5.93 | 51.6 | Glod4 Isoform 3 of Glyoxalase domain-containing protein 4 | 3 | -25.5% | -43.5% | -18.0% |
| 81 | 4.23 | 35.3 | Rab1b Ras-related protein Rab-1B | 2 | -25.5% | -79.1% | -53.6% |
| 82 | 2 | 21.2 | Apeh Isoform 1 of Acylamino-acid-releasing enzyme | 1 | -25.5% | 72.2% | 97.7% |
| 83 | 4.94 | 22.8 | Cs Citrate synthase, mitochondrial precursor | 2 | -24.8% | -84.0% | -59.2% |
| 84 | 2.12 | 7.7 | Plcd1 Phospholipase C delta-1 | 1 | -24.8% | -0.9% | 23.9% |
| 85 | 7.54 | 39.8 | H2afy Isoform 2 of Core histone macro-H2A.1 | 5 | -24.1% | 15.9% | 40.0% |
| 86 | 2.96 | 29.2 | Rab11a Ras-related protein Rab-11A | 1 | -24.1% | -72.7% | -48.6% |
| 87 | 1.7 | 23.1 | Me1 malic enzyme 1, NADP(+)-dependent, cytosolic | 1 | -24.1% | 36.8% | 60.9% |
| 88 | 4.38 | 17.2 | Scp2 Isoform SCPx of Non-specific lipid-transfer protein | 1 | -23.4% | -37.5% | -14.0% |
| 89 | 3.04 | 23.8 | Epb4.1 Isoform 1 of Protein 4.1 | 1 | -23.4% | -40.3% | -16.9% |
| 90 | 2.1 | 20.7 | Dctn2 Dynactin subunit 2 | 1 | -23.4% | -75.1% | -51.7% |
| 91 | 1.36 | 14.3 | Nap1l1 Nucleosome assembly protein 1-like 1 | 0 | -23.4% | 7.7% | 31.1% |
|  |  |  |  | average | -42.7% | -14.0% | 28.7% |

Supplementary table 1B. The differentially expressed proteins up-regulated by 1.3-fold identified by proteomic analysis.

| N | Unused Protscore | %Cov | Protein Name | Peptides (95%) | Untreated vs. normal | Treated vs. normal | Effect of treatment |
| --- | --- | --- | --- | --- | --- | --- | --- |
|  |  |  |  |  | (% change) | (% change) | (% change) |
| 1 | 26.14 | 30.3 | Hspg2 perlecan | 12 | 30.6% | -34.5% | -65.2% |
| 2 | 3.86 | 47 | Spr sepiapterin reductase | 2 | 30.6% | 12.7% | -17.9% |
| 3 | 3.23 | 20 | LOC100046594 similar to heterogeneous nuclear ribonucleoprotein U-like 2 | 1 | 30.6% | -20.6% | -51.2% |
| 4 | 2 | 50.8 | Prr6 Isoform 1 of Proline-rich protein 6 | 1 | 30.6% | 22.5% | -8.2% |
| 5 | 17.02 | 38.1 | Myh11 Myh11 protein | 11 | 31.8% | 97.7% | 65.9% |
| 6 | 10.32 | 60.8 | Des Desmin | 8 | 33.1% | -19.8% | -52.9% |
| 7 | 2.34 | 23.7 | Snx2 Sorting nexin-2 | 1 | 33.1% | 12.7% | -20.3% |
| 8 | 23.13 | 68.3 | Mdh2 Malate dehydrogenase, mitochondrial precursor | 14 | 35.5% | -32.1% | -67.6% |
| 9 | 12.47 | 69.7 | Hnrnpa2b1 43 kDa protein | 5 | 35.5% | -44.5% | -80.1% |
| 10 | 4.82 | 21.7 | Fh1 Isoform Mitochondrial of Fumarate hydratase, mitochondrial precursor | 2 | 35.5% | -42.5% | -78.0% |
| 11 | 1.7 | 22.7 | Hsd17b4 Peroxisomal multifunctional enzyme type 2 | 1 | 35.5% | -50.8% | -86.3% |
| 12 | 4.19 | 34.2 | Slc25a4 ADP/ATP translocase 1 | 2 | 36.8% | -0.9% | -37.7% |
| 13 | 6.2 | 38.7 | Hsd17b10 hydroxyacyl-Coenzyme A dehydrogenase type II | 3 | 38.0% | -23.4% | -61.5% |
| 14 | 7.22 | 56.2 | Sept2 Septin-2 | 4 | 39.3% | 85.4% | 46.0% |
| 15 | 2.39 | 49.5 | Rpl26 Ribosomal protein L26 | 1 | 39.3% | 253.2% | 213.9% |
| 16 | 51.17 | 44.8 | Spnb2 Isoform 1 of Spectrin beta chain, brain 1 | 26 | 40.6% | -28.9% | -69.5% |
| 17 | 5.67 | 55.2 | Tpm4 Tropomyosin alpha-4 chain | 6 | 40.6% | 80.3% | 39.7% |
| 18 | 2.46 | 39.8 | Rab2b Ras-related protein Rab-2B | 1 | 40.6% | -28.9% | -69.5% |
| 19 | 4.31 | 47.8 | LOC100048238;LOC100039820;LOC100044132 similar to ribosomal protein S28 | 1 | 41.9% | -55.1% | -97.0% |
| 20 | 1.65 | 14.7 | Hadh Hydroxyacyl-coenzyme A dehydrogenase, mitochondrial precursor | 1 | 41.9% | -56.8% | -98.7% |
| 21 | 1.45 | 32.7 | Cct7 T-complex protein 1 subunit eta | 0 | 41.9% | -63.0% | -104.9% |
| 22 | 6 | 16.7 | Usp5 2 days neonate thymus thymic cells cDNA, RIKEN full-length enriched library, clone:E430021I10 product:Ubiquitin carboxyl-terminal hydrolase 5 (EC 3.1.2.15) (Ubiquitin thiolesterase 5) (Ubiquitin- specific processing protease 5) (Deubiquitinating enzyme 5) (Isopeptidase T) homolog | 3 | 43.2% | -74.7% | -117.9% |
| 23 | 2.73 | 42.3 | Cotl1 Coactosin-like protein | 1 | 43.2% | -20.6% | -63.8% |
| 24 | 21.86 | 25.8 | Fbn1 Mutant fibrillin-1 | 7 | 47.2% | -32.7% | -79.9% |
| 25 | 6 | 53.6 | Pfn1 Profilin-1 | 3 | 47.2% | -77.7% | -125.0% |
| 26 | 4.13 | 38.4 | Fabp3 Fatty acid-binding protein, heart | 2 | 47.2% | 51.4% | 4.1% |
| 27 | 1.7 | 25.5 | Ap2a1 Isoform B of AP-2 complex subunit alpha-1 | 1 | 47.2% | -13.7% | -60.9% |
| 28 | 2.03 | 31.2 | Rplp0 60S acidic ribosomal protein P0 | 1 | 48.6% | -44.5% | -93.1% |
| 29 | 9.25 | 42.4 | Actn4 Alpha-actinin-4 | 10 | 50.0% | -63.7% | -113.7% |
| 30 | 2.02 | 59.7 | Eif4h Isoform Long of Eukaryotic translation initiation factor 4H | 1 | 50.0% | 231.1% | 181.2% |
| 31 | 6.18 | 37.3 | Arhgdia Rho GDP-dissociation inhibitor 1 | 3 | 51.4% | -23.4% | -74.8% |
| 32 | 3.15 | 24.8 | Hpx hemopexin | 1 | 51.4% | -48.0% | -99.4% |
| 33 | 48.59 | 77 | Vim Vimentin | 29 | 52.8% | -43.5% | -96.3% |
| 34 | 3.3 | 7.7 | Anpep Aminopeptidase N | 2 | 52.8% | -64.4% | -117.1% |
| 35 | 2 | 17.1 | Ccdc128 Isoform 1 of Coiled-coil domain-containing protein 128 | 1 | 54.2% | 172.9% | 118.7% |
| 36 | 2 | 20.5 | Zadh1 Isoform 1 of Zinc-binding alcohol dehydrogenase domain-containing protein 1 | 2 | 54.2% | -9.6% | -63.8% |
| 37 | 2.81 | 36 | Psmc3 Proteasome (Prosome, macropain) 26S subunit ATPase 3 | 1 | 57.0% | 19.1% | -37.9% |
| 38 | 2.79 | 29.2 | Mapk3 Mitogen-activated protein kinase 3 | 1 | 58.5% | 50.0% | -8.5% |
| 39 | 18.72 | 68.3 | Col6a3 Collagen alpha3(VI) precursor (Fragment) | 42 | 60.0% | -55.1% | -115.1% |
| 40 | 4.97 | 58.7 | D10Jhu81e;LOC100046684 ES1 protein homolog, mitochondrial precursor | 3 | 60.0% | 92.3% | 32.4% |
| 41 | 2.26 | 27 | EG665033 Collagen alpha-5 | 1 | 60.0% | 22.5% | -37.5% |
| 42 | 2 | 25 | Crip2 Cysteine-rich protein 2 | 1 | 60.0% | 29.4% | -30.5% |
| 43 | 10.74 | 46.8 | Eno3 Beta-enolase | 8 | 61.4% | 22.5% | -39.0% |
| 44 | 4.96 | 33.7 | Cald1 89 kDa protein | 2 | 61.4% | 193.8% | 132.3% |
| 45 | 2.97 | 35.3 | Tnni2 Troponin I, skeletal, fast 2 | 2 | 67.5% | -48.5% | -116.0% |
| 46 | 2.17 | 42.3 | Anxa7 annexin A7 | 1 | 67.5% | 39.3% | -28.2% |
| 47 | 19.31 | 47.3 | Anxa5 Annexin A5 | 10 | 69.0% | -69.5% | -138.6% |
| 48 | 9.7 | 54.3 | Dscaml1 Down syndrome cell adhesion molecule-like protein (Fragment) | 5 | 69.0% | 23.6% | -45.5% |
| 49 | 9.93 | 53 | Got2 Aspartate aminotransferase, mitochondrial precursor | 5 | 70.6% | -62.0% | -132.6% |
| 50 | 7.01 | 38.8 | Tnnt3 Troponin T3, skeletal, fast | 2 | 77.0% | 142.1% | 65.1% |
| 51 | 2.06 | 22.8 | Gpnmb glycoprotein (transmembrane) nmb | 1 | 85.4% | -25.5% | -110.9% |
| 52 | 24.65 | 54.8 | LOC675857 similar to valosin isoform 1 | 11 | 88.8% | -44.5% | -133.3% |
| 53 | 15.88 | 21.2 | Ttn Isoform 1 of Titin | 6 | 92.3% | -21.3% | -113.6% |
| 54 | 12.78 | 40.9 | Plec1 Isoform PLEC-0 of Plectin-1 | 6 | 95.9% | 2.8% | -93.1% |
| 55 | 24.25 | 71.8 | Tpm1 Isoform 1 of Tropomyosin alpha-1 chain | 11 | 99.5% | 58.5% | -41.0% |
| 56 | 8.69 | 37.5 | Aldh3a1 aldehyde dehydrogenase family 3, subfamily A1 | 4 | 99.5% | 231.1% | 131.6% |
| 57 | 39.04 | 68.7 | Hspa5 78 kDa glucose-regulated protein precursor | 20 | 101.4% | 94.1% | -7.3% |
| 58 | 6.4 | 22.1 | Srl Isoform 1 of Sarcalumenin precursor | 3 | 101.4% | 50.0% | -51.4% |
| 59 | 38.42 | 83.9 | Lmna Isoform A of Lamin-A/C | 23 | 103.2% | -28.2% | -131.5% |
| 60 | 2 | 19.1 | Ugdh UDP-glucose 6-dehydrogenase | 1 | 110.9% | 6.7% | -104.2% |
| 61 | 54.5 | 72.1 | Myh4 Myosin-4 | 77 | 129.1% | 39.3% | -89.8% |
| 62 | 12.38 | 56.4 | Ckm Creatine kinase M-type | 6 | 142.1% | 39.3% | -102.8% |
| 63 | 3.41 | 17.6 | Nnt NAD(P) transhydrogenase, mitochondrial precursor | 2 | 144.3% | 64.4% | -79.9% |
| 64 | 2.68 | 43.1 | Tnnc2 Troponin C, skeletal muscle | 1 | 148.9% | -88.8% | -237.7% |
| 65 | 1.52 | 13 | Crip1 Cysteine-rich protein 1 | 1 | 158.2% | 392.0% | 233.8% |
| 66 | 9.24 | 72.5 | Tpm2 Isoform 1 of Tropomyosin beta chain | 10 | 180.5% | -46.5% | -227.1% |
| 67 | 24.52 | 45.3 | Atp2a1 Sarcoplasmic/endoplasmic reticulum calcium ATPase 1 | 13 | 196.5% | -53.9% | -250.4% |
| 68 | 148.57 | 70.6 | Myh1 Myosin-1 | 79 | 213.3% | 107.0% | -106.3% |
| 69 | 10.86 | 26.9 | Thbs1 Thrombospondin 1 | 5 | 283.7% | 146.6% | -137.1% |
| 70 | 15.58 | 63.2 | Myh2 Myosin, heavy polypeptide 2, skeletal muscle, adult | 63 | 508.1% | 332.5% | -175.6% |
| 71 | 12.43 | 38.6 | Actn2 actinin alpha 2 | 6 | 687.1% | 470.2% | -216.9% |
|  |  |  |  | average | 86.9% | 30.0% | -57.0% |
